# Prevalence of and risk factors for *Plasmodium* spp. co-infection with hepatitis B virus: a systematic review and meta-analysis

**DOI:** 10.1186/s12936-020-03428-w

**Published:** 2020-10-15

**Authors:** Kwuntida Uthaisar Kotepui, Manas Kotepui

**Affiliations:** Medical Technology, School of Allied Health Sciences, Walailak University, Tha Sala, Nakhon Si Thammarat, Thailand

**Keywords:** Malaria, *Plasmodium*, Hepatitis B virus, HBV, Co-infection

## Abstract

**Background:**

*Plasmodium* spp. and hepatitis B virus (HBV) are among the most common infectious diseases in underdeveloped countries. This study aimed to determine the prevalence of *Plasmodium* spp. and HBV co-infection in people living in endemic areas of both diseases and to assess the risk factors related to this co-infection.

**Methods:**

The PubMed, Web of Science, and Scopus databases were searched. Observational cross-sectional studies and retrospective studies assessing the prevalence of *Plasmodium* species and HBV co-infection were examined. The methodological quality of the included studies was assessed with the Newcastle-Ottawa Scale (NOS), a tool for assessing the quality of nonrandomized studies in meta-analyses, and heterogeneity among the included studies was assessed with Cochran's *Q* test and the I^2^ (inconsistency) statistic. The pooled prevalence of the co-infection and its 95% confidence interval (CI) were estimated using the random-effects model, depending on the amount of heterogeneity there was among the included studies. The pooled odds ratio (OR) represented the difference in qualitative variables, whereas the pooled mean difference (MD) represented the difference in quantitative variables. Meta-analyses of the potential risk factors for *Plasmodium* spp. and HBV co-infection, including patient age and gender, were identified and represented as pooled odds ratios (OR) and 95% CIs. Publication bias among the included studies was assessed by visual inspection of a funnel plot to search for asymmetry.

**Results:**

Twenty-two studies were included in the present systematic review and meta-analysis. Overall, the pooled prevalence estimate of *Plasmodium* spp. and HBV co-infection was 6% (95% CI 4–7%, Cochran's *Q* statistic < 0.001, I^2^: 95.8%), with prevalences of 10% in Gambia (95% CI: 8–12%, weight: 4.95%), 8% in Italy (95% CI 5–12%, weight: 3.8%), 7% in Nigeria (95% CI 4–10%, weight: 53.5%), and 4% in Brazil (95% CI 2–5%, weight: 19.9%). The pooled prevalence estimate of *Plasmodium* spp. and HBV co-infection was higher in studies published before 2015 (7%, 95% CI 4–9%, Cochran's *Q* statistic < 0.001, I^2^: 96%) than in those published since 2015 (3%, 95% CI 1–5%, Cochran's *Q* statistic < 0.001, I^2^: 81.3%). No difference in age and risk of *Plasmodium* spp. and HBV co-infection group was found between the *Plasmodium* spp. and HBV co-infection and the *Plasmodium* monoinfection group (p: 0.48, OR: 1.33, 95% CI 0.60–2.96). No difference in gender and risk of *Plasmodium* spp. and HBV co-infection group was found between the *Plasmodium* spp. and HBV co-infection and HBV co-infection group and the *Plasmodium* monoinfection group (p: 0.09, OR: 2.79, 95% CI 0.86–9.10). No differences in mean aspartate aminotransferase (AST), mean alanine aminotransferase (ALT), or mean total bilirubin levels were found (p > 0.05) between the *Plasmodium* spp. and HBV co-infection group and the *Plasmodium* monoinfection group.

**Conclusions:**

The present study revealed the prevalence of *Plasmodium* spp. and HBV co-infection, which will help in understanding co-infection and designing treatment strategies. Future studies assessing the interaction between *Plasmodium* spp. and HBV are recommended.

## Background

Malaria in humans is caused by the infection of at least one of the five *Plasmodium* species, including *Plasmodium falciparum*, *Plasmodium vivax*, *Plasmodium ovale*, *Plasmodium malariae,* and *Plasmodium knowlesi* [1]. Malaria is transmitted by the bite of an infected female *Anopheles* mosquito [2]. Malaria remains endemic among people around the world, especially people in the World Health Organization (WHO) African Region and the WHO South-East Asia Region [3]. According to the latest World Malaria Report 2019, there were an estimated 228 million cases of malaria with an estimated number of malaria deaths at 405,000, mostly children under 5 years of age (67%) [4]. Most malaria cases were reported from six countries in the African region including Nigeria (25%), the Democratic Republic of the Congo (12%), Uganda (5%), Côte d'Ivoire (4%), Mozambique (4%) and Niger (4%) [4]. Hepatitis B infection is caused by hepatitis B virus (HBV), a double-stranded DNA virus belonging to the *Hepadenaviridae* family, and its infection leads to a wide range of clinical spectra from acute to chronic hepatitis, cirrhosis, and hepatocellular carcinoma [5]. The infection of HBV in individuals was acquired through exposure to potentially infectious blood or blood products or through percutaneous exposure to sharp contamination [6]. Globally, an estimated 257 million people were living with chronic HBV infection in 2015, and most people in the WHO African Region and the Western Pacific Region were affected [7]. The reduction in HBV prevalence among children (1.3%) was seen after the introduction of the hepatitis B vaccine in 2015 [7].

*Plasmodium* spp. and HBV infections are endemic among people residing in the same regions and are prone to co-infect individuals because of their geographical coincidence [8, 9]. In individuals with *Plasmodium* spp. and HBV co-infection, the two pathogens use the liver as their host during their developmental stages, which may result in liver injuries and lead to increased mortality and morbidity [10]. A previous study demonstrated that HBV infections could reduce the ability of the liver to kill *Plasmodium* parasites [10, 11]. A study in patients with *Plasmodium* spp. and HBV co-infection demonstrated that *P. falciparum* modulates HBV viremia in patients with chronic HBV infection [12]. A study conducted in Asia proposed that chronic HBV infection may lead to the synergistic multiplication of *P. falciparum* malaria, but the overall risk of death was not significantly higher in coinfected patients [13]. Another study proposed that HBV infection might reduce the density of *Plasmodium* parasites in malaria patients with no organ dysfunction [9], while a previous study demonstrated that *Plasmodium* and HBV co-infection significantly increased the density of malaria parasites [14].

Currently, studies on the risk factors for *Plasmodium* spp. and HBV co-infection and the differences in liver function tests (LFTs) are sparse and inconsistent. Demographic profiles of patients, including age and gender, might explain the possible risks for co-infection, as suggested by previous studies indicating that most cases of *Plasmodium* spp. and HBV co-infection occured in patients aged 20–50 years [15, 16], while another study demonstrated a higher proportion of *Plasmodium* spp. and HBV co-infection cases among patients who are ≥ 50 years of age [17]. A better understanding of the overall prevalence of *Plasmodium* spp. and HBV co-infection, the potential risk factors, and the LFTs in infected individuals could help endemic countries to diagnose, prevent, and control *Plasmodium* spp. and HBV co-infection. Therefore, the present study aimed to synthesize evidence regarding *Plasmodium* spp. and HBV co-infection using a meta-analytic approach and to assess the effects of demographic profiles, including age and gender, on the risks of *Plasmodium* spp. and HBV co-infection. In addition, the differences in the levels of LFTs between individuals with *Plasmodium* spp. and HBV co-infection and those with *Plasmodium* spp. monoinfection were also investigated.

## Methods

### Protocol and registration

This study was conducted in accordance with the Preferred Reporting Items for Systematic Reviews and Meta-analyses (PRISMA) guidelines, which provide an evidence-based minimum set of items for reporting in systematic reviews and meta-analyses (PRISMA Checklist S1) [18]. The protocol was registered at the International Prospective Register of Systematic Reviews (PROSPERO) with a registration number: CRD42020196790. *Plasmodium* spp. infections were diagnosed by a standard method, microscopy or other alternative methods, including polymerase chain reaction (PCR), rapid diagnosis test (RDT), or a combination of those methods. HBV infections were diagnosed by RDT or PCR, enzyme-linked immunosorbent assay (ELISA), or a combination of those methods.

### Search strategies

The PubMed, Web of Science, and Scopus databases were searched for studies on co-infection with *Plasmodium* spp. and HBV that were published prior to April 16, 2020. To maximize the search, only three Medical Subject Headings (MeSH) were used: (malaria OR plasmodium) AND "hepatitis B" AND "co-infection". To maximize the number of included studies, studies on humans published in any language were considered. The references of the included studies and relevant review articles were also reviewed to identify additional relevant studies.

### Eligibility criteria and selection of studies

Studies were included in this study if they were (1) observational studies performed on participants coinfected with *Plasmodium* spp. and HBV and (2) observational studies reporting on the number of *Plasmodium* spp. and HBV coinfected and monoinfected participants. Studies that did not meet the inclusion criteria were excluded, including animal studies, books and book chapters, experimental studies, studies on malaria co-infection with other pathogens, studies that did not examine co-infection, case reports/case series, randomized control trials, studies with a similar group of participants, and studies with no full text available. The relevant review articles were stored and reviewed as potentially eligible articles that met the inclusion criteria only, but they were not included in the meta-analysis.

### Data extraction

Two authors (MK and KUK) independently screened and selected the relevant studies related to the eligibility criteria. Data from the potentially eligible studies were retrieved and extracted to a standardized data extraction form (Microsoft Excel, Microsoft Corporation, USA). Any discrepancies in study selection and extractions were resolved by consensus or discussion. Information on authors, year of publication, study area, years of the study conducted, study design, age range, gender, number of *Plasmodium* spp. monoinfections, number of HBV monoinfections, number of *Plasmodium* spp. and HBV co-infection, detection methods for *Plasmodium* spp. and HBV, and laboratory data (LFTs) were extracted.

### Quality of the included studies

The quality of the included studies was determined following the Newcastle-Ottawa Scale (NOS), a tool for assessing the quality of nonrandomized studies in meta-analyses that consists of three domains: selection, comparability, and outcomes [19]. A maximum of six stars (the highest quality) for the cross-sectional and retrospective studies was modified from the NOS to assessing the quality of the included studies. Any studies rated ≥ 5 stars were considered high-quality studies, while studies rated < 5 stars were considered low-quality studies (Table 2).

### Data synthesis

The primary outcome of the present study was the pooled prevalence of *Plasmodium* spp. and HBV co-infection, which was estimated using the Freeman–Tukey double arcsine transformation method with the DerSimonian and Laird random-effects model. These analyses were run using the “metaprop” command provided in Stata (StataCorp, USA) [20, 21]. In 2015, the WHO released the ‘WHO Guidelines for the treatment of malaria’ [22], which consisted of recommendations on the diagnosis and treatment of severe and uncomplicated malaria among at-risk populations [22]. The subgroup analysis of the pooled prevalence estimate between years of studies conducted (year 2014 or less, year 2015 and beyond) was performed to identify any differences in the pooled prevalence estimate between subgroups. The secondary outcome of the present study was the pooled effect estimates (odds ratio, OR) for the age group, gender, aspartate aminotransferase (AST) levels, alanine aminotransferase (ALT) levels, and total bilirubin levels. Differences in the effect estimates between the groups were examined. The results of the pooled effect estimates were presented as a pooled OR and its 95% confidence interval (CI) for dichotomous variables, whereas the results of the pooled effect estimates were presented as pooled mean difference (MD) and its 95% CI for continuous variables. The heterogeneity across the included studies was assessed with Cochran's *Q* test and Higgins I^2^ (inconsistency) statistic. A significant Cochran's *Q* test (p < 0.05) with an I^2^ value greater than 50% indicated substantial heterogeneity. The fixed-effects model was used in cases of no significant heterogeneity, while the random-effects model was used in cases of significant heterogeneity across the included studies.

### Publication bias assessment

The publication bias among the included studies was assessed by a visual inspection of the funnel plot to search for asymmetry (the asymmetrical distribution of the included studies in the graph between the OR and SE (logOR)). The funnel plot was generated with data on the age of participants and status of *Plasmodium* spp. and HBV co-infection.

## Results

### Characteristics of the included studies

**Fig. 1:**
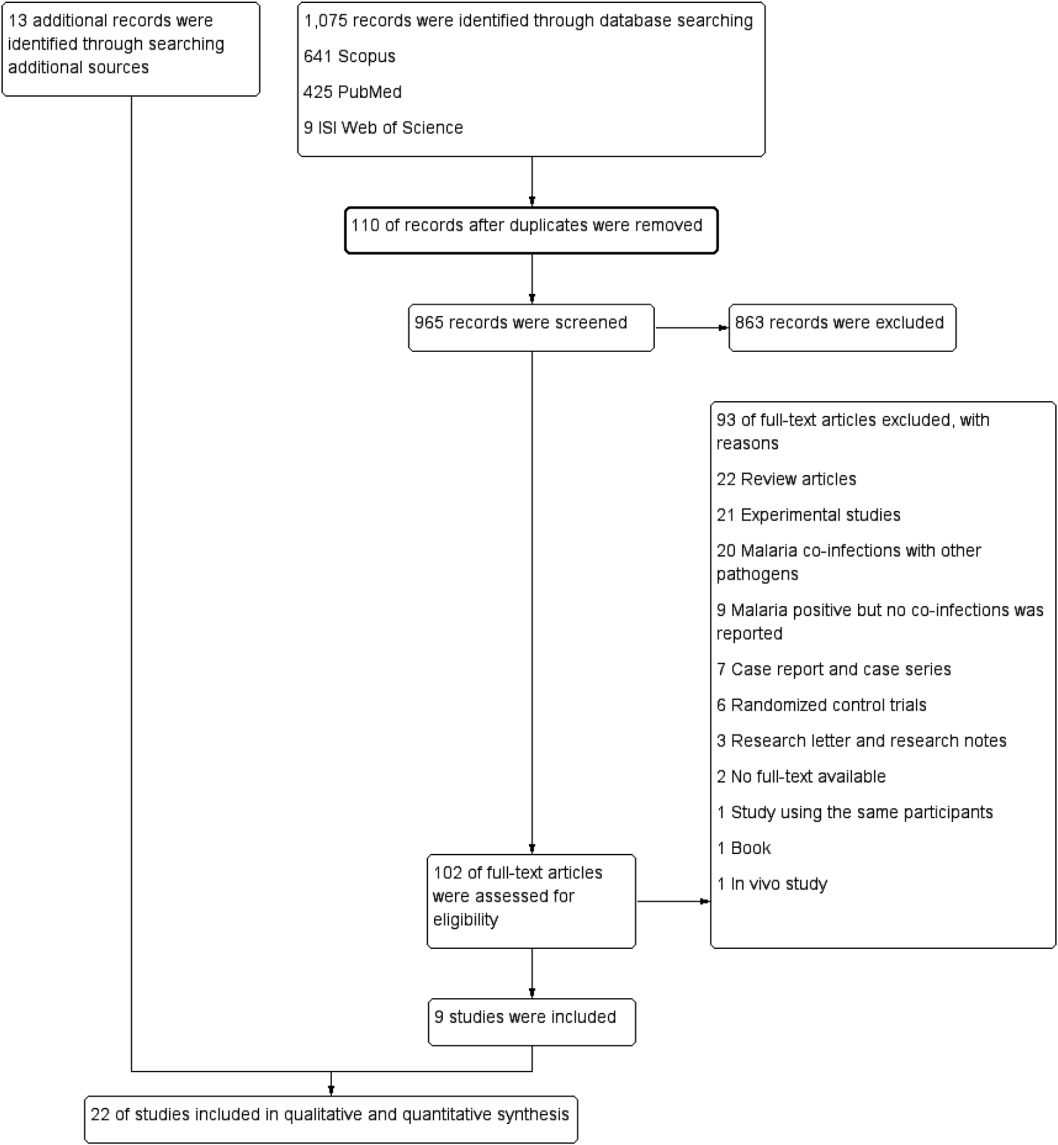
Flow chart for the study selection

The process of the present systematic review is demonstrated in the study flow diagram (Fig. 1). The initial search yielded 1,075 articles, and the titles and abstracts of 965 articles were screened. The full texts of 102 articles that met the inclusion criteria were screened. Finally, 9 studies [8–10, 23–28] were included in the quantitative synthesis. Thirteen additional studies [11, 14–17, 29–36] were identified and included after reviewing the references of the nine included studies and additional searches of other databases. The main characteristics of the 22 included studies [8–11, 14–17, 23–36] are presented in Table 1. Of the 22 included studies, 13 studies [9–11, 14, 16, 17, 24, 26, 28, 31, 32, 34, 35] were conducted before 2015, while three studies [23, 29, 30] were conducted in or after 2015. One study was conducted during 2013–2015, while five studies [8, 15, 27, 33, 36] did not specify the year of study conducted. Among the 22 included studies, approximately half of the studies (12/22; 54.5%) were carried out in Nigeria [10, 15, 16, 26, 28–31, 33–36], while the remaining 10 studies were carried out in Brazil [9, 14, 17, 32], Ghana [8, 23, 25], Central African Republic [24], Italy [27], and The Gambia [11]. Twenty of the 22 included studies used cross-sectional designs, while two studies used retrospective designs. The age range of participants was reported in 20 studies, whereas 2 studies did not report the age range of their participants. Approximately half of the included studies [8–10, 14–16, 24, 26–28, 30, 32, 33, 35, 36] (15/22; 68.2%) performed studies on both men and women, while four studies [23, 25, 29, 31] performed studies only in females. The sex of the participants was not specified in two studies [11, 17]. Five studies [9, 26, 30, 35, 36] examined patients seeking care at hospitals, five studies examined pregnant women [23, 25, 29, 31, 34], three studies [16, 28, 33] examined febrile patients, one study examined blood donors [10], one study examined residents [17], one study examined malaria patients [32], one study examined patients attending health check-ups [15], one study examined transfusion recipients [8], one study examined patients who tested negative for yellow fever [24], one study examined African immigrants [27], one study examined children [11], and one study enrolled patients with asymptomatic/symptomatic *P. vivax*, patients with HBV infection/co-infection, and healthy individuals [14]. Ten studies [10, 11, 15, 16, 28–31, 35, 36] used only standard microscopy, five studies [24–26, 33, 34] used only RDT, two studies [8, 27] used only PCR, two studies [8, 27] used both microscopy and PCR, one study [23] used both RDT and PCR, and one study [17] used ELISA for the detection of malaria parasites. The detection methods for *Plasmodium* spp. and HBV and the number of patients with *Plasmodium* spp. monoinfection and *Plasmodium* spp. and HBV co-infection are shown in Table 1.

**Table 1:** Characteristics of the included studies

No	Author, year	Study area (years of the conducted)	Study design	Age range	Sex	Participants	No. positive either malaria or hepatitis B	Malaria monoinfection	Detection method for *Plasmodium* spp.	Hepatitis B monoinfection (HBsAg)	Detection method for HBV	Co-infection
1	Abah and Udoidang 2019 [30]	Nigeria (2018)	Cross-sectional study	15–70 years < 20 (92) 21–30 (136) 31–40 (149) 41–50 (119) ≥ 51 (104)	Male (309) Female (291)	OPD patients (600)	308 < 20 (52), 21–30 (106), 31–40 (67), 41–50 (42), ≥ 51 (41)	246 < 20 (40), 21–30 (76), 31–40 (53), 41–50 (38), ≥ 51 (39)	Microscopy	36 < 20 (8), 21–30 (16), 31–40 (9), 41–50 (2), ≥ 51 (1)	RDT	26 < 20 (4), 21–30 (14), 31–40 (5), 41–50 (2), ≥ 51 (1)
2	Abah et al. 2019 [29]	Nigeria (2016)	Cross-sectional study	15–24 (59), 25–34 (120), 35–44 (75), 45–54 (46)	Female	Pregnant women (300)	137 15–24 (42), 25–34 (53), 35–44 (27), 45–54 (15)	110 15–24 (27), 25–34 (46), 35–44 (23), 45–54 (14)	Microscopy	17 15–24 (10), 25–34 (5), 35–44 (2), 45–54 (0)	RDT	10 15–24 (5), 25–34 (2), 35–44 (2), 45–54 (1)
3	Adeleke et al. 2013 [31]	Nigeria (2011–2012)	Cross-sectional study	15–20 (10), 21–25 (65), 26–30 (80), 31–35 (40), > 35 (5)	Female	Pregnant women (200)	34 15–20 (2), 21–25 (8), 26–30 (18), 31–35 (4), > 35 (0)	26 15–20 (1), 21–25 (5), 26–30 (16), 31v35 (4), > 35 (0)	Microscopy	6 15–20 (1), 21–25 (3), 26–30 (2), 31–35 (0), > 35 (0)	RDT	2
4	Aernan et al. 2011 [10]	Nigeria (2009)	Cross-sectional study	18–22 (87), 23–27 (97), 28–32 (96), 33–37 (28), 38–42 (24), 43–47 (4), 48–52 (4)	Male (229) Female (108)	Blood donor (337)	Not specified	Not specified	Microscopy	Not specified	RDT	137 18–22 (40), 23–27 (44), 28–32 (22), 33–37 (16), 38–42 (11), 43–47 (2), 48–52 (2)
5	Afolabi et al. 2018 [15]	Nigeria	Cross-sectional study	1–10 (8), 11–20 (87), 21–30 (307), 31–40 (55), 41–50 (25), > 50 (18)	Male (167) Female (333)	Health check-up (500)	436	385 1–10 (6), 11–20 (79), 21–30 (215), 31–40 (51), 41–50 (19), > 50 (15)	Microscopy	31	RDT	20 1–10 (0), 11–20 (0), 21–30 (3), 31–40 (8), 41–50 (4), > 50 (3)
6	Anabire et al. 2019 [23]	Ghana (2016–2017)	Cross-sectional study	Uninfected (28 ± 5.8), Pf 27.4 ± 5.7, HBV (27.2 ± 4.8), co-infections (27.1 ± 5.6)	Female	Pregnant women (2071)	469	Pf (278)	RDT, PCR	115	RDT	36
7	Andrade et al. 2011 [9]	Brazil (2006–2007)	Cross-sectional study	5–70 years 5–15 (40), 16–30 (152), 31–59 (326), ≥ 60 (62)	Male (267) Female (369)	OPD patients (636)	392	335 Pv (363), Pf(56), mixed (12)	Microscopy, PCR	29	ELISA, Real-time PCR	28
8	Braga et al. 2005 [17]	Brazil (2000)	Cross-sectional study	< 1 (18), 2–4 (94), 5–14 (182), 15–29 (158), 30–49 (98), ≥ 50 (55)	Not specified	Residents (605)	342	311 < 1 (4), 2–4 (30), 5–14 (74), 15–29 (92), 30–49 (68), ≥ 50 (43)	ELISA	20 < 1 (0), 2–4 (0), 5–14 (5), 15–29 (9), 30–49 (6), ≥ 50 (0)	ELISA	11 < 1 (0), 2–4 (0), 5–14 (3), 15–29 (5), 30–49 (3), ≥ 50 (0)
9	Braga et al. 2006 [32]	Brazil (2001–2002)	Cross-sectional study	< 14 years	Male (410) Female (369)	Malaria patients (545)	545	Pv (333), Pf (193)	ELISA	Not specified	ELISA	23
10	Cruz et al. 2019 [14]	Brazil (2006–2007)	Retrospective study	Not specified	Male (267) Female (334)	Asymptomatic *P. vivax*(145), Symptomatic *P. vivax* (179), HBV co-infections (28), HBV (29), Healthy (165)	381	Pv (324)	Microscopy, PCR	29	ELISA	28
11	Dabo et al. 2015 [16]	Nigeria (2013)	Cross-sectional study	15–64 15–24 (67), 25–34 (75), 35–44 (37), 45–54 (12), 55–64 (9)	Male (90) Female (110)	Febrile patients (200)	73	51 15–24 (24), 25–34 (13), 35–44 (6), 45–54 (6), 55–64 (2)	Microscopy	13 15–24 (4), 25–34 (4), 35–44 (4), 45–54 (0), 55–64 (1)	ELISA	9 15–24 (2), 25–34 (5), 35–44 (2), 45–54 (0), 55–64 (0)
12	Freimanis et al. 2012 [8]	Ghana	Cross-sectional study	< 20 (9), 20–29 (44), 30–39 (36), 40–49 (13), ≥ 50 (14)	Male (14) Female (103)	Transfusion recipients (117)	75	33 Pf (52), Pf/Pm (5), Pf/Po (1)	PCR	17	RDT, EIA, PCR	25
13	Gadia et al. 2017 [24]	Central African Republic (2008–2010)	Retrospective study	< 15 (35), 16–24 (24), 25–34 (21), ≥ 35 (17)	Male (43) Female (54)	Patients who tested negative for yellow fever IgM (162), for Pf (198), for HBV (162)	40	Pf (4)	RDT	32	ELISA	4
14	Helegbe et al. 2018 [25]	Ghana (2013–2015)	Cross-sectional study	15–19 (72), 20–24 (622), 25–29 (1,143), 30–34 (874), 35–39 (354), ≥ 40 (62)	Female	Pregnant women (3,127)	471	339	RDT	109	RDT	23
15	Kolawole and Kana 2018 [33]	Nigeria	Cross-sectional study	18–25 years	Male (72) Female (128)	Febrile patients (200)	111	62	RDT	38	RDT, EIA	11
16	Omalu et al. 2012 [34]	Nigeria (2011)	Cross-sectional study	Not specified		323 Pregnant women (259), nonpregnant (64)	297	267 (Pregnant 216, non-pregnant 51)	RDT	30	RDT	27 (Pregnant 21, non-pregnant 6)
17	Oyeyemi et al. 2015 [26]	Nigeria (2014)	Cross-sectional study	4–73 years 4–12 (3), 13–21 (38), 22–30 (49), 31–39 (43), ≥ 40 (33)	Male (66) Female (100)	OPD patients (166)	Not specified	Pf (44) 4–12 (3), 13–21 (17), 22–30 (9), 31–39 (13), ≥ 40 (2)	RDT	27 4–12 (0), 13–21 (9), 22–30 (10), 31–39 (4), ≥ 40 (4)	RDT	11 4–12 (0), 13–21 (5), 22–30 (1), 31–39 (3), ≥ 40 (2)
18	Scotto and Fazio 2018 [27]	Italy	Cross-sectional study	16–40 years	Male (162) Female (33)	African immigrants (195)	103	62 Pf (24)	PCR	26	RDT, PCR	15
19	Sharif et al. 2015 [28]	Nigeria (2013)	Cross-sectional study	15–24 (67), 25–34 (75), 35–44 (37), 45–54 (12), 55–64 (9)	Male (90) Female (110)	Febrile patients (200)	30	15 15–24 (6), 25–34 (7), 35–44 (2), 45–54 (0), 55–64 (0)	Microscopy	9 15–24 (2), 25–34 (3), 35–44 (3), 45–54 (0), 55–64 (1)	ELISA	6 15–24 (1), 25–34 (3), 35–44 (2), 45–54 (0), 55–64 (0)
20	Thursz et al. 1995 [11]	Gambia (1988–1990)	Case–control study	Children	Not specified	Children (1,268): malaria (750), nonmalaria (518)	929	750 Severe malaria (414), mild malaria (336)	Microscopy	55	ELISA	124
21	Wokem and Amacree 2018 [35]	Nigeria (2013)	Cross-sectional study	Malaria (189): ranged 0–5, 6–11 HBV (36): ranged 24–29, 30–35	Male Female	OPD patients (700)	238	189	Microscopy	36	ELISA	13
22	Yohanna et al. 2016 [36]	Nigeria	Cross-sectional study	< 15 (458), > 15 (286)	Male Female	OPD patients (750)	532	399	Microscopy	87	RDT	46

*OPD* out patients department, *RDT* rapid diagnostic test, *PCR* polymerase chain reaction, *ELISA* enzyme-linked immunosorbent assay

### The prevalence of *Plasmodium* spp. and HBV co-infection

**Fig. 2:**
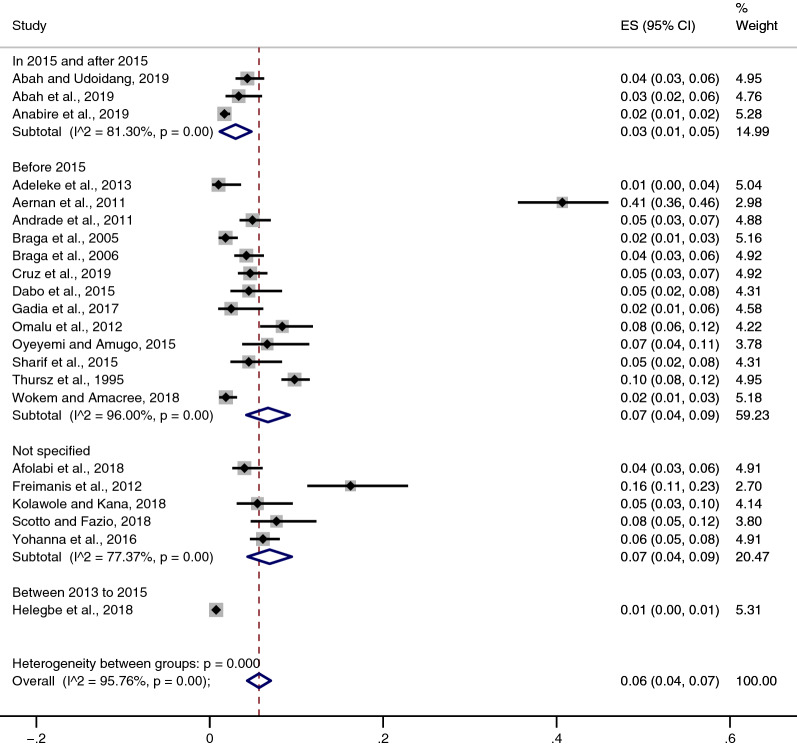
Pooled prevalence of *Plasmodium* spp. and HBV co-infection

All 22 studies included in the present systematic review and meta-analysis provided data on the prevalence of *Plasmodium* spp. and HBV co-infection. The results showed that the prevalence estimate of *Plasmodium* spp. and HBV co-infection among 22 studies varied widely, ranging between 1 and 41%. The highest proportion of *Plasmodium* spp. and HBV co-infection was found in Nigerian blood donors (41%) as reported by Aernan et al. [10]. Overall, the pooled prevalence estimate of *Plasmodium* spp. and HBV co-infection was 6% (95% CI 4–7%, Cochran's *Q* statistic < 0.001, I^2^: 95.8%), and there was highly significant heterogeneity (p < 0.001, I^2^: 95.8%) among the included studies (Fig. 2).

### Subgroup analysis on the prevalence of *Plasmodium* spp. and HBV co-infection

A subgroup analysis of the pooled prevalence estimate between studies conducted before 2015 and studies conducted since 2015 revealed that the pooled prevalence estimate of *Plasmodium* spp. and HBV co-infection in 13 studies published before 2015 was 7% (95% CI 4–9%, Cochran's *Q* statistic < 0.001, I^2^: 96%), while the pooled prevalence estimate of *Plasmodium* spp. and HBV co-infection in three studies published since 2015 was 3% (95% CI 1–5%, Cochran's *Q* statistic < 0.001, I^2^: 81%). The pooled prevalence estimate of *Plasmodium* spp. and HBV co-infection in studies conducted during 2013–2015 and studies that did not specify the year of studies conducted are demonstrated in Fig. 2.

**Fig. 3:**
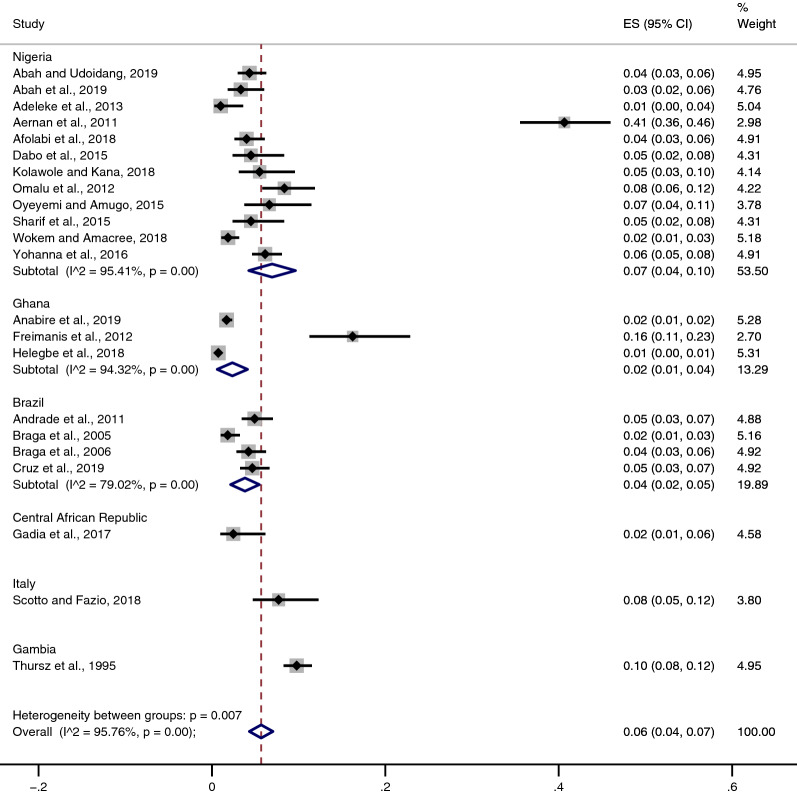
Pooled prevalence of *Plasmodium* spp. and HBV co-infection by countries

Subgroup analysis of the pooled prevalence of *Plasmodium* spp. and HBV co-infection between studies conducted in different countries was also analysed. The results showed that the pooled prevalence estimate of *Plasmodium* spp. and HBV co-infection was 8% in Italy (95% CI 5–12%), 7% in Nigeria (95% CI 4–10%, I^2^: 95.41%), 4% in Brazil (95% CI 2–5%, I^2^: 79.02%), 2% in Ghana (95% CI 1–1%, I^2^: 94.32%), 2% in the Central African Republic (95% CI 1–6%), and 1% in Gambia (95% CI 8–12%) (Fig. 3).

### Differences in age, gender and potential risk of *Plasmodium* spp. and HBV co-infection

**Fig. 4:**
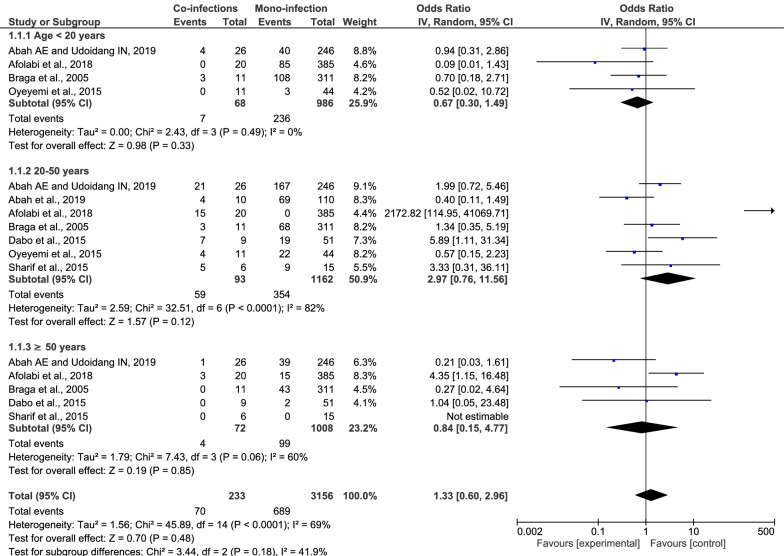
Difference in age between *Plasmodium* spp. and HBV co-infection and *Plasmodium* spp. monoinfection

The identified studies were classified into subgroups by age and gender (Additional file 1: Table S1). Among the seven included studies, there was no difference in the age of individuals between the *Plasmodium* spp. and HBV co-infection group and the *Plasmodium* spp. monoinfection group (p: 0.18, OR: 1.33, 95% CI 0.6–0.96, Cochran's *Q* statistic < 0.0001, I^2^: 69%). Subgroup analysis of age groups (< 20, 20–50, and ≥ 50 years) was performed to identify any difference in age and risk of *Plasmodium* spp. and HBV co-infection. The results showed that no significant difference in age and risk of *Plasmodium* spp. and HBV co-infection was found in participants aged 20–50 years (p: 0.12, OR: 2.97, 95% CI 0.76–11.56), < 20 years (p: 0.33, R: 0.67, 95% CI 0.30–1.49), and ≥ 50 years (p: 0.85, OR: 0.84, 95% CI 0.15–4.77) (Fig. 4).

**Fig. 5:**
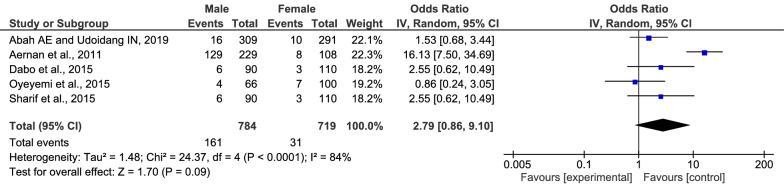
Difference in gender between *Plasmodium* spp. and HBV co-infection and *Plasmodium* spp. monoinfection

An analysis of gender and risk of *Plasmodium* spp. and HBV co-infection was performed. The results showed that among the five included studies, there was no significant difference in gender and risk of *Plasmodium* spp. and HBV co-infection (p: 0.09, OR: 2.79, 95% CI 0.86–9.10, I^2^: 84%) (Fig. 5). The highest proportion of males (OR: 16.1, 95% CI 7.5–34.7) was found in blood donors as reported by Aernan et al*.* [10].

### Differences in LFTs between *Plasmodium* spp. and HBV co-infection and *Plasmodium* spp. monoinfection

**Fig. 6:**
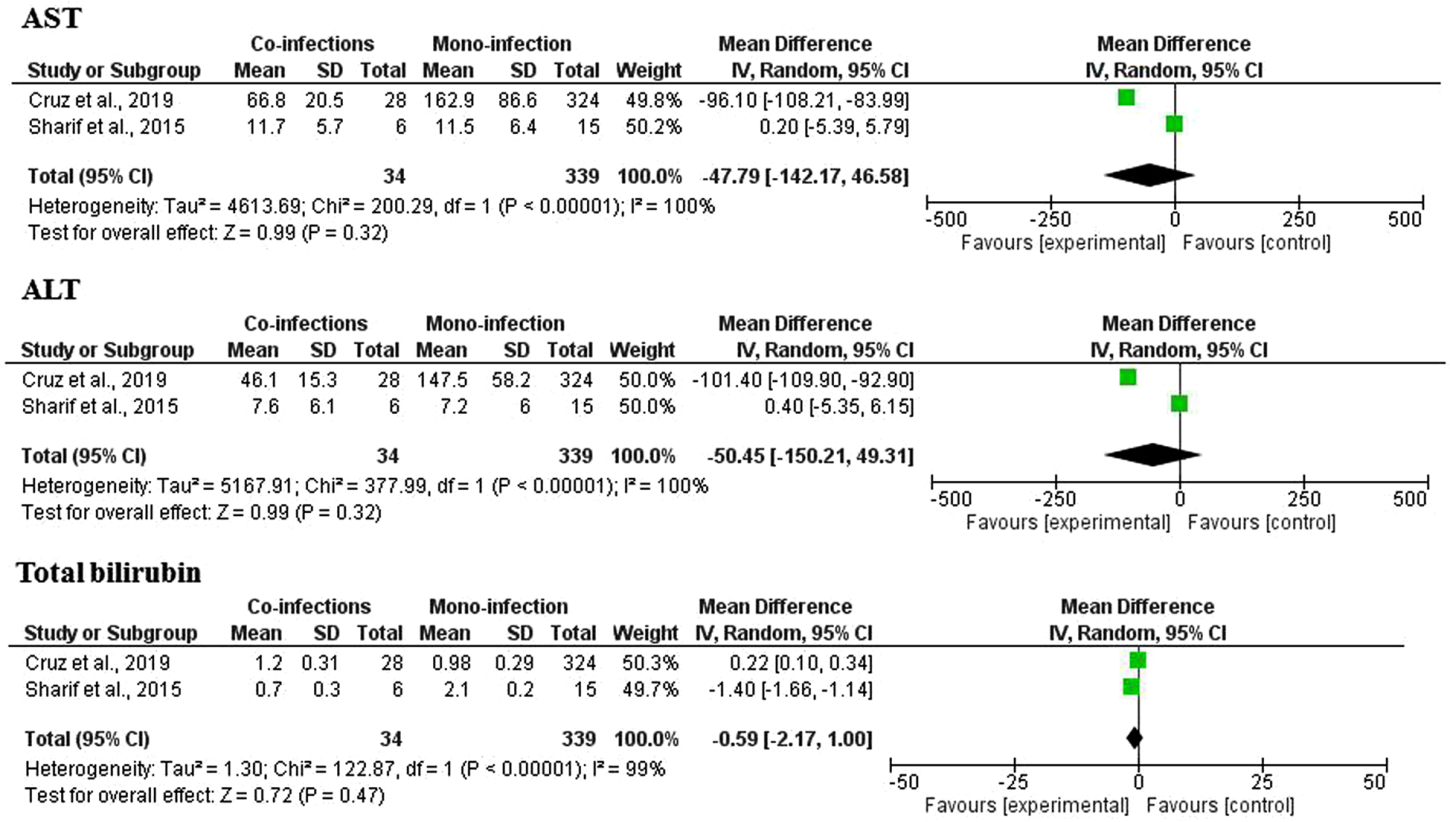
Differences in AST, ALT, and total bilirubin levels between *Plasmodium* spp. and HBV co-infection and *Plasmodium* spp. monoinfection

Only two studies [14, 28] reported the AST, ALT, and total bilirubin levels and were included in the meta-analysis of mean differences (Additional file 2: Table S2). The results showed that no significant difference in AST levels was found between the *Plasmodium* spp. and HBV co-infection group and the *Plasmodium* spp. monoinfection group (p: 0.32, MD: -47.8, 95% CI -142.2–46.6, Cochran's *Q* statistic < 0.00001, I^2^: 100%). No significant difference in ALT levels was found between the *Plasmodium* spp. and HBV co-infection group and the *Plasmodium* spp. monoinfection group (p: 0.32, OR: -50.5, 95% CI -150.2–49.3, Cochran's *Q* statistic < 0.00001, I^2^: 100%). No significant difference in total bilirubin levels was found between the *Plasmodium* spp. and HBV co-infection group and the *Plasmodium* spp. monoinfection group (p: 0.47, MD: -0.59, 95% CI -2.17–1.0, Cochran's *Q* statistic < 0.00001, I^2^: 99%) (Fig. 6).

### Quality of the included studies

Overall, the quality of the 22 included studies was assessed using the NOS with some modifications for cross-sectional and retrospective studies. All included studies were high-quality and had ratings ranging from six stars to seven stars (Table 2).

**Table 2:** Quality of the included studies

No	References	Selection				Compatibility	Exposure			Total score (7)	Rating (High, moderate, low quality)
		Is the case definition adequate?	Representativeness of the cases	Selection of controls	Definition of controls		Ascertainment of exposure	Same method of ascertainment for cases and controls	Non-response rate		
1	Abah and Udoidang 2019 [30]			NA	NA					7	High
2	Abah et al. 2019 [29]			NA	NA					6	High
3	Adeleke et al. 2013 [31]			NA	NA					6	High
4	Aernan et al. 2011 [10]			NA	NA					7	High
5	Afolabi et al. 2018 [15]			NA	NA					7	High
6	Anabire et al. 2019 [23]			NA	NA					6	High
7	Andrade et al. 2011 [9]			NA	NA					7	High
8	Braga et al. 2005 [17]			NA	NA					7	High
9	Braga et al. 2006 [32]			NA	NA					7	High
10	Cruz et al. 2019 [14]			NA	NA					6	High
11	Dabo et al. 2015 [16]			NA	NA					7	High
12	Freimanis et al. 2012 [8]			NA	NA					6	High
13	Gadia et al. 2017 [24]			NA	NA					6	High
14	Helegbe et al. 2018 [25]			NA	NA					6	High
15	Kolawole and Kana 2018 [33]			NA	NA					7	High
16	Omalu et al. 2012 [34]			NA	NA					6	High
17	Oyeyemi et al. 2015 [26]			NA	NA					7	High
18	Scotto and Fazio 2018 [27]			NA	NA					7	High
19	Sharif et al. 2015 [28]			NA	NA					7	High
20	Thursz et al. 1995 [11]			NA	NA					6	High
21	Wokem and Amacree 2018 [35]			NA	NA					7	High
22	Yohanna et al. 2016 [36]			NA	NA					7	High

A star system developed by the Newcastle–Ottawa Scale (NOS) for assessing the quality of non-randomized studies in meta-analyses, which consists of three domains including selection, comparability, and outcomes. A maximum of six stars (the highest quality) for the cross-sectional and retrospective studies was modified from the NOS for assessing the quality of the included studies. Any study rated ≥ 5 stars was considered a high-quality study

*NA* Not assessed

### Publication bias

**Fig. 7:**
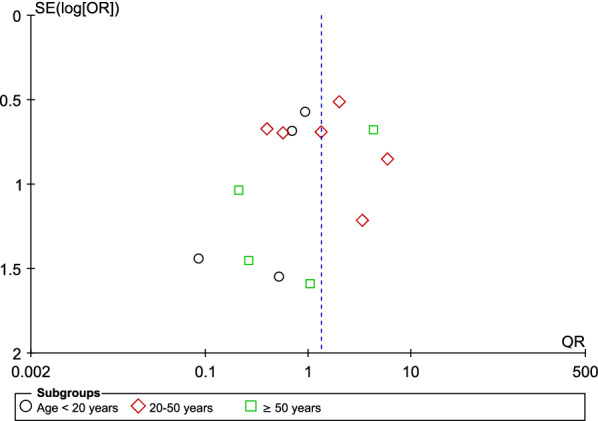
Funnel plot

The funnel plot revealed an asymmetrical distribution of the included studies in the graph between the OR and SE (logOR) (Fig. 7), indicating publication bias due to the small study effects.

## Discussion

The present systematic review and meta-analysis provided information on the overall prevalence of *Plasmodium* spp. and HBV co-infection. The results demonstrated that there was a high prevalence estimate of *Plasmodium* spp. and HBV co-infection (6%). Nevertheless, the pooled prevalence estimate of *Plasmodium* spp. and HBV co-infection was not precisely estimated, as indicated by the highly significant amount of heterogeneity among the included studies (I^2^ = 95.76, p < 0.001). When assessing the individual studies, *Plasmodium* spp. and HBV co-infection was prevalent in most tropical and sub-Saharan African countries, particularly Nigeria [10, 15, 16, 26, 28–31, 33–36], Ghana [8, 23, 25], Central African Republic [24], and The Gambia [11]. The pooled prevalence estimate of *Plasmodium* spp. and HBV co-infection was highest (6%) among the included studies conducted in Nigeria. However, due to the significant amount of heterogeneity (p < 0.001, I^2^: 95.41%) across these included studies, the pooled prevalence estimate in Nigeria might be confounded by at least one study conducted in Nigerian blood donors, which reported the highest proportion of *Plasmodium* spp. and HBV co-infection (41%) [10]. Compared with studies conducted in Nigeria, the pooled prevalence estimate of *Plasmodium* spp. and HBV co-infection in Ghana was lower in the included studies (2%). There was also a significant amount of heterogeneity in the pooled subgroup analysis (p < 0.001, I^2^: 94.32%), which might be due to at least one study reporting a prevalence of 16% among transfusion recipients [8]. The pooled prevalence estimate of *Plasmodium* spp. and HBV co-infection in studies conducted in Brazil showed the lowest amount of heterogeneity (p < 0.001, I^2^: 79.02%) compared with studies conducted in Nigeria and Ghana. The study conducted among African immigrants in Italy reported a high prevalence of *Plasmodium* spp. and HBV co-infection and suggested that this finding was due to the participants having acquired semi-immunity with submicroscopic levels of parasitaemia, resulting in no sign or symptoms of malaria before detection [27].

The subgroup analysis of the years of studies revealed that the pooled prevalence estimate of *Plasmodium* spp. and HBV co-infection was higher among studies conducted before 2015 (7%) than those conducted since 2015 (3%). However, the pooled prevalence of *Plasmodium* spp. and HBV co-infection among studies conducted before 2015 was not precisely estimated, as indicated by the significant amount of heterogeneity among the 13 included studies [9–11, 14, 16, 17, 24, 26, 28, 31, 32, 34, 35] (p < 0.001, I^2^: 96%). The pooled prevalence of *Plasmodium* spp. and HBV co-infection among studies conducted since 2015 (3%) was lower than the prevalence among studied conducted before 2015. In addition, a significantly lower amount of heterogeneity among the three studies [23, 29, 30] (p < 0.001, I^2^: 81.3%) was found, indicating a more reliable pooled prevalence estimate of *Plasmodium* spp. and HBV co-infection than the prevalence among the studies conducted before 2015. In light of these results, the reduction in the pooled prevalence between studies conducted in and after 2015 compared with studies conducted before 2015 might be due to the new WHO guidelines implemented in 2015, which updated the recommendations for the treatment of malaria, including the dosing of drugs in children and the use of drugs for preventing malaria in high-risk groups (young children, pregnant women, tuberculosis or HIV/AIDS patients, non-immune travellers) [22].

Little is known about the effect of *Plasmodium* spp. and HBV co-infection on disease severity. The interaction between two pathogens in hosts has been studied. The interactions between *Plasmodium* spp. and HBV in the same individuals in Papua New Guinea demonstrated that patients with severe malaria had the lowest prevalence of HBV infection [37]. The study of the immune response among patients with malaria and HBV co-infection demonstrated a robust pro-inflammatory Type 1 immune response (Th1), which is important for *Plasmodium* spp. clearance induced by HBV; nevertheless, it caused disease severity from co-infection [38]. HBV replication in liver cells also enhanced the production of interferon (IFN)-γ and IFN-α/β. A study in experimental mice demonstrated that intrahepatic HBV replication was inhibited by the *Plasmodium yoelii* 17X NL [39]. They also reported that *Plasmodium* spp. and HBV coinfected individuals demonstrated elevated concentrations of IL-10 (eight-fold) and C–C Motif Chemokine Ligand 2 (CCL2) in comparison to those with *P. vivax* infection [14]. CCL2 has been reported to be produced by hepatocytes during HBV infection [40] and is related to *P. vivax* infection [41].

A meta-analysis was conducted to determine whether age was a potential risk factor for *Plasmodium* spp. and HBV co-infection. The meta-analysis of age included seven studies [15–17, 26, 28–30] and revealed that no significant difference in age was found between the *Plasmodium* spp. and HBV co-infection group and the *Plasmodium* spp. monoinfection group. There was a significant heterogeneity among the included studies (p < 0.0001, I^2^: 69%). To explore the source of heterogeneity among the included studies that might affect the pooled OR estimate, a subgroup analysis of age (< 20, 20–50, and > 50 years of age) was performed. The subgroup analysis demonstrated that no significant difference in age or risk of co-infection was found among the three age groups. The pooled OR estimates were reliable in individuals aged < 20 (p < 0.49, I^2^: 0%) and those aged > 50 years (p: 0.06, I^2^: 60%) with no significant amount of heterogeneity among the included studies. This finding implies that an age < 20 and an age > 50 years were not potential risk factors for *Plasmodium* spp. and HBV co-infection, while the pooled OR estimates of age were not reliable in individuals aged between 20–50 years, as indicated by the significant amount of heterogeneity among the included studies (p < 0.0001, I^2^: 82%). This means that an age between 20 and 50 years old might be a potential risk factor for *Plasmodium* spp. and HBV co-infection, but this finding could not be confirmed in the present meta-analysis and needs to be evaluated by further longitudinal studies. In light of these results, research conducted in individuals aged between 20–50 years by two studies, Afolabi et al*.* [15] and Dabo et al*.* [16], demonstrated the highest prevalences of *Plasmodium* spp. and HBV co-infection in their studies. The higher prevalence of *Plasmodium* spp. and HBV co-infection among the youthful age group could be attributed to their higher-risk behaviours for infection by both *Plasmodium* spp. and HBV, including unprotected sexual activities, drug abuse, fashionable tattooing and skin piercing, the lack of mosquito nets for beds, and failure to complete the recommended drug doses for treatments [10, 30]. Another possible explanation for the high proportion of *Plasmodium* spp. and HBV co-infection among the youthful age group could be explained by females in this age group giving birth to children. These females (pregnant) visited clinics or hospitals during their pregnancy health check-up, which resulted in a diagnosis and contributed to the high prevalence of *Plasmodium* spp. and HBV co-infection. This rationale was supported by the high proportion of pregnant women coinfected with *Plasmodium* spp. and HBV in four of the included studies [23, 26, 29, 31].

The meta-analysis of gender among five included studies [10, 16, 26, 28, 30] demonstrated that male and female participants were comparable in risk of *Plasmodium* spp. and HBV co-infection (p: 0.09) with a significant amount of heterogeneity across the included studies (p < 0.0001, I^2^: 84%). This means that the pooled OR of gender in those five studies was not precisely estimated due to the high heterogeneity. In light of this result, the association between gender and risk of *Plasmodium* spp. and HBV co-infection should be further investigated, as the high proportion of males coinfected with *Plasmodium* spp. and HBV than females was reported in the study by Aernan et al*.* [30].

The differences in LFTs between *Plasmodium* spp. and HBV co-infection were performed using two studies [14, 28]. The results demonstrated that no significance in any of the three LFTs, including AST, ALT, and total bilirubin levels, was found (p > 0.05); there was a significant amount of heterogeneity among these studies (p < 0.0001, I^2^ ≥ 99%). Considering individual studies in the meta-analysis, one study demonstrated lower ALT and AST levels in the *Plasmodium* spp. and HBV co-infection group than in the *Plasmodium* spp. monoinfection group [14], while another study demonstrated no difference in the ALT and AST levels between groups [28]. The included 2018 study by Kolawole and Kana indicated high ALT (54.5%, 6/11 cases) and total bilirubin levels (72.7%, 8/11 cases) among patients with *Plasmodium* spp. and HBV co-infection [33].

The present study had several limitations. First, there was a limited number of included studies reporting on the prevalence of, potential risk factors for, and LFTs among individuals with *Plasmodium* spp. and HBV co-infection and in comparison with *Plasmodium* spp. monoinfection. Therefore, the meta-analyses of the pooled prevalences, pooled ORs, and pooled MDs were limited by these data. Second, there was a significant amount of heterogeneity among the included studies, resulting in imprecise estimates of the pooled prevalence estimate of *Plasmodium* spp. and HBV co-infection. Therefore, the pooled prevalence estimates should be interpreted with caution. Third, the present study did not analyse the other potential risk factors for *Plasmodium* spp. and HBV co-infection since most of the included studies did not examine or report for these factors.

## Conclusion

The present study demonstrated the status of *Plasmodium* spp. and HBV co-infection. The results of this study can be used to support health care communities, helping them to recognize the double burden of these two infections, control *Plasmodium* spp. parasites and provide regular HBV vaccinations.

## Supplementary information

**Additional file 1: Table S1.** Age groups of *Plasmodium* spp. and HBV co-infection and monoinfection.

**Additional file 2: Table S2.** Laboratory parameters in *Plasmodium* spp. and HBV co-infection and monoinfection.

## Data Availability

The datasets used during the current study are available without restriction.

## Abbreviations

ALT: Alanine aminotransferase
ANC: Antenatal care
AST: Aspartate aminotransferase
CCL2: C–C Motif Chemokine Ligand 2
CI: Confidence interval
HBV: Hepatitis B virus
IFN: Interferon
LFTs: Liver function tests
NOS: Newcastle–Ottawa Scale
OR: Odds ratio
PCR: Polymerase chain reaction
PRISMA: Preferred Reporting Items for Systematic Reviews and Meta-analyses
RDT: Rapid diagnosis test
MD: Mean difference
Th1: Type 1 immune response
WHO: World Health Organization
