# Supplementary material for: Prevalence of and risk factors for Plasmodium spp. co-infection with hepatitis B virus: a systematic review and meta-analysis
Source: Malar J. 2020 Oct 15;19:368. doi: 10.1186/s12936-020-03428-w (PMC7560023; doi:10.1186/s12936-020-03428-w)
Supplement: Supplementary file 1 — Additional file 1: Table S1. Age groups of Plasmodium spp. and HBV co-infection and monoinfection. [file 12936_2020_3428_MOESM1_ESM.docx]

**Table S1** Age groups of coinfection and monoinfection

| No. | Author, year | Male | | Female | |
| --- | --- | --- | --- | --- | --- |
|  |  | Examined (n) | Infected (n) | Examined (n) | Infected (n) |
| 1. | Abah AE and Udoidang IN, 2019 | 309  <20 (36)  21 – 30 (58)  31 – 40 (78)  41 – 50 (62)  ≥ 51 (75) | 16  <20 (3)  21 – 30 (9)  31 – 40 (2)  41 – 50 (1)  ≥ 51 (1) | 291  <20 (56)  21 – 30 (78)  31 – 40 (71)  41 – 50 (57)  ≥ 51 (29) | 10  <20 (1)  21 – 30 (5)  31 – 40 (3)  41 – 50 (1)  ≥ 51 (0) |
| 4. | Aernan et al., 2011 | 229 | 129  18 – 22 (38),  23 – 27 (43),  28 – 32 (22),  33 – 37 (14),  38 – 42 (10),  43 – 47 (0),  48 – 52 (2) | 108 | 8  18 – 22 (2),  23 – 27 (1),  28 – 32 (0),  33 – 37 (2),  38 – 42 (1),  43 – 47 (2),  48 – 52 (0) |
| 11. | Dabo et al., 2015 | 90 | 6  15 – 24 (1),  25 – 34 (3),  35 – 44 (2),  45 – 54 (0),  55 – 64 (0) | 110 | 3  15 – 24 (1),  25 – 34 (2),  35 – 44 (0),  45 – 54 (0),  55 – 64 (0) |
| 17. | Oyeyemi et al., 2015 | 66 | 4 | 100 | 7 |
| 19. | Sharif et al., 2015 | 90 | 6  15 – 24 (1),  25 – 34 (3),  35 – 44 (2),  45 – 54 (0),  55 – 64 (0) | 110 | 3  15 – 24 (1),  25 – 34 (2),  35 – 44 (0),  45 – 54 (0),  55 – 64 (0) |
