# Supplementary material for: Prevalence of and risk factors for Plasmodium spp. co-infection with hepatitis B virus: a systematic review and meta-analysis
Source: Malar J. 2020 Oct 15;19:368. doi: 10.1186/s12936-020-03428-w (PMC7560023; doi:10.1186/s12936-020-03428-w)
Supplement: Supplementary file 2 — Additional file 2: Table S2. Laboratory parameters in Plasmodium spp. and HBV co-infection and monoinfection. [file 12936_2020_3428_MOESM2_ESM.docx]

**Table S2** Laboratory parameters in coinfection and monoinfection

| No. | Author, year | AST (IU/L) | | ALT (IU/L) | | Total bilirubin (mg/dL) | |
| --- | --- | --- | --- | --- | --- | --- | --- |
|  |  | Coinfections | Malaria monoinfections | Coinfections | Malaria monoinfections | Coinfections | Malaria monoinfections |
| 10. | Cruz et al., 2019 | 65.45 (32.75–103.6), 66.8±20.5 | 114.8 (61.06-361.1),  162.9±86.6 | 42.75 (23.05–75.98), 46.1±15.3 | 114.9 (79.3-280.75), 147.5±58.2 | 1.20 (0.80–1.60), 1.2±0.31 | 0.95 (0.65-1.38), 0.98±0.29 |
| 19. | Sharif et al., 2015 | 11.7±5.7 | 11.5±6.4 | 7.6±6.1 | 7.2±6.0 | 0.7±0.3 | 2.1±0.2 |
